# Chicago Face Database: Multiracial expansion

**DOI:** 10.3758/s13428-020-01482-5

**Published:** 2020-10-09

**Authors:** Debbie S. Ma, Justin Kantner, Bernd Wittenbrink

**Affiliations:** 1 Department of Psychology, California State University, Northridge, 18111 Nordhoff Street, Northridge, CA 91330-8255 USA; 2 University of Chicago Booth School of Business, Chicago, IL USA

**Keywords:** Biracial, Multiracial, Face perception, Racial categorization, Face database

## Abstract

Multiracial individuals represent a growing segment of the population and have been increasingly the focus of empirical study. Much of this research centers on the perception and racial categorization of multiracial individuals. The current paper reviews some of this research and describes the different types of stimuli that have been used in these paradigms. We describe the strengths and weaknesses associated with different operationalizations of multiracialism and highlight the dearth of research using faces of real multiracial individuals, which we posit may be due to the lack of available stimuli. Our research seeks to satisfy this need by providing a free set of high-resolution, standardized images featuring 88 real multiracial individuals along with extensive norming data and objective physical measures of these faces. These data are offered as an extension of the widely used Chicago Face Database and are available for download at www.chicagofaces.org for use in research.

## Introduction

The prevalence of individuals who identify as multiracial in the United States has risen steadily over the past several decades. The U.S. Census Bureau reports that individuals indicating more than one race rose from 6.8 million in 2000 to 9 million in 2010; by 2050, 1 in 5 Americans is expected to identify as multiracial (Farley, 2001). As these figures rise, it has become increasingly important to understand how multiracials are perceived by others. Studying multiracials raises important and novel theoretical questions for social psychologists interested in social categorization, trait ascription, and evaluation, as well as for cognitive psychologists, who have had a longstanding interest in the mental representation of categories and category learning. Moreover, understanding how individuals from this potentially ambiguous category are perceived and classified may provide insight into their status in and their interactions with American society (Albuja, Sanchez, & Gaither, 2018; Bratter & Gorman, 2011; Remedios & Chasteen, 2013).

Accordingly, researchers have begun to turn their attention to investigations of multiracials in recent years. Two broad streams constitute extant research about multiracials. The first seeks to understand the lived experience of multiracial individuals (for a review see Gaither, 2015), while the second centers on the perception of multiracial individuals by others. The current research focuses on the latter issue and contributes to the growing literature on multiracial face perception by offering a free set of normed facial stimuli of self-identified multiracial individuals. Additionally, because so little is known about the properties of multiracial faces, we explore some of the physical and subjective features that contribute to perceptions of multiracialism. In the next section, we discuss various resources currently used to operationalize multiracialism in face perception research.

## Current operationalizations of multiracialism

Multiracialism is often operationalized in terms of ancestry. For example, Remedios and Chasteen (2013) selected participants for their studies based on whether they reported to have parents who belonged to different races (see also Gaither, Sommers, & Ambady, 2013; Townsend, Fryberg, Wilkins, & Markus, 2012). Likewise, ancestry is the criterion adopted by the US Census for categorizing an individual as multiracial, allowing Census respondents to check more than one box for racial ancestry. The use of ancestry in determining multiracial status suggests – somewhat misleadingly – an objective and verifiable criterion. There is no a priori biological standard for determining an ancestor’s race. General consensus among biologists and genetics researchers holds that race is not a functional concept for classifying humans (e.g., Serre & Pääbo, 2004; Yudell, Roberts, DeSalle, & Tishkoff, 2016). Instead, racial ancestry reports will have to rely on either knowledge of the ancestors’ self-identification, or on one’s awareness that the ancestors were labeled or treated by others as belonging to a particular race – knowledge respondents may or may not have.

Circumventing some of these issues, researchers have alternatively asked respondents directly to indicate whether they consider themselves to be of “mixed race” (Binning, Unzueta, Huo, & Molina, 2009; Udry, Li, & Hendrickson-Smith, 2003). Defining multiracial through self-identification is widely used in the identity literature, which focuses more on the experience (rather than perception) of multiracialism (Phinney & Alipuria, 2006). For example, within the developmental literature, researchers administer measures of ethnic identity to assess the extent to which an individual feels they belong to, identify with, or engage with different racial or ethnic groups, as is the case with the Multigroup Ethnic Identity Measure (Phinney, 1992; Spencer, Icard, Harachi, Catalano, & Oxford, 2000). Conceptualizing multiracialism in this way may also offer a distinct advantage over defining multiracialism strictly based on ancestry, because research shows that multiracial individuals consider their racial identities as fluid and can change their racial identification across time (Harris & Sim, 2002; Hitlin, Scott Brown, & Elder, 2006).

By contrast, research on the perception of multiracial individuals by others more commonly relies on social consensus to determine whether a person is multiracial. By this standard, a person is considered multiracial if others conceive of the target as such (Gaither, Chen, Pauker, & Sommers, 2019). Social consensus could be based on either ancestry or physical appearance (Chen, 2019). With respect to physical appearance, social consensus may be determined by whether a target possesses features that are associated with a particular race (e.g., skin tone, eye color, etc.; Ma, Kantner, Dunn, & Benitez, 2020). For example, perceivers may expect that black–white biracials have freckles and learn to associate this feature with this category. It is worth noting that in the field, very few stimuli reach high levels of social consensus in race categorization data. In our own work, biracial faces are categorized as such with as low as 20% agreement (Ma, Kantner, Benitez, & Dunn, 2020; Ma, Kantner, Dunn, et al., 2020) and an average of 60% across raters would be considered fairly high. That said, social consensus may be less elusive in places where there are more multiracial residents, such as in Hawaii (Pauker, Carpinella, Lick, Sanchez, & Johnson, 2018). As multiracial populations continue to grow, it will be interesting to track whether social consensus also increases.

As an alternative to social consensus, some researchers operationalize multiracialism using racially ambiguous targets. As an example, Pauker et al. (2009) asked 26 participants to categorize 500 racially ambiguous faces as either Black or White, which led them to select 40 faces that were perceived as White and Black equally. This strategy may be problematic, however, because a maximally racially ambiguous face could be perceived as always White by one subset of raters and always Black by another subset, but the data are often collapsed at the target level, which could obscure such a pattern and undermine the goal of selecting faces that are at the cusp of being one race or another. Additionally, defining multiracialism in terms of racial ambiguity is problematic because even racially ambiguous faces are not always perceived as multiracial (Chen & Hamilton, 2012; Chen, Pauker, Gaither, Hamilton, & Sherman, 2018; Ho Kteily, & Chen, 2020). Moreover, just as social perception research demonstrates wide variability among monoracial faces (Ma, Correll, & Wittenbrink, 2015; Strom, Zebrowitz, Zhang, Bronstad, & Lee, 2012), multiracial faces possess significant featural variation that may or may not encompass racial ambiguity (Chen, Norman, & Nam, 2020).

Clearly, multiracialism can be conceptualized based on a number of dimensions (Chen, 2019; Roth, 2016) and some have even proposed that ancestry, self-identification, and social perception should all be considered when assessing multiracial status (Woo, Austin, Williams, & Bennett, 2011). Given the many conceptions of multiracialism, it should not come as a surprise that this multifaceted variable has also be operationalized in a variety of ways. Within the multiracial perception literature alone, biracialism/multiracialism has been operationalized using images of real multiracial faces, facial morphs, computer-generated faces, and racial background information. In what follows, we review some of these operationalizations and discuss the respective strengths and weaknesses of each.

### Racial background information

One technique for operationalizing multiracialism in this literature does not involve presenting participants with any visual stimuli at all and instead asks participants to provide categorical judgments based on background information regarding a target’s racial makeup. For example, Ho, Sidanius, Levin, and Banaji (2011; Study 1) asked participants to imagine a child who was half or one-quarter Asian (or Black) and one half or three-quarters White and indicate whether they considered the hypothetical target person to be mostly Asian (or Black) or mostly White. In follow-up studies (Studies 2A and 2B), Ho et al. (2011) participants were presented with family trees in which the racial background of a target individual’s grandparents was manipulated and were asked to judge the target’s racial background. Operationalizing multiracialism by presenting ancestral information is straightforward and provides insights into perceivers’ internal mental representations of multiracial individuals without adulteration from the experimenter. This technique is also consistent with research examining the psychology of being multiracial. At the same time, the method is somewhat limited in scope. For example, it cannot tell us how multiracial individuals are perceived in many real-world situations where faces (and not the family tree information) constitute the only available information. As such, some researchers have supplemented racial background information by simultaneously presenting participants with facial stimuli (e.g., Peery & Bodenhausen, 2008; Skinner & Nicolas, 2015).

### Artificial faces

A second class of operationalizations of multiracialism involves the use of artificial face stimuli. These include facial morphs of real monoracials as well as computer-generated faces. Facial morphs are produced using software that mathematically combines two inputs, generally referred to as “parent” faces, which researchers assume possess the features that define multiracial faces. For example, a researcher seeking to produce an Asian–White multiracial stimulus would first select an image of a monoracial Asian face and a monoracial White face. Next, the researcher would select corresponding landmarks on each of the monoracial faces (e.g., setting matched-paired points at the center of each target’s pupil, the left edge of the nose, the right edge of the mouth, etc.), which are used to derive a mathematical map of the faces through Euclidean algebra. Finally, the researcher can determine the “racial composition” of the resulting stimulus face by adjusting the contribution of each parent face. Most typically, researchers use mathematical averages of monoracial parents (e.g., 50% Asian, 50% White) for biracial stimuli.

Facial morphing is a common operationalization for multiracialism in the literature (e.g., Peery & Bodenhausen, 2008) and has obvious appeal. First, as we address below, there is a general dearth of real biracial face stimuli available and facial morphing offers a convenient and inexpensive means of creating large numbers of stimuli. Second, morphing software affords tight control over the stimuli – researchers can select parent faces that meet desired specifications (e.g., particular racial backgrounds, attractiveness levels, age, masculinity, etc.) and can titrate the amount each parent contributes to the stimuli. For example, even though researchers traditionally use average faces (e.g., 50% Black, 50% White) for biracial stimuli, the researcher could select a target anywhere along the morphed continuum (e.g., 16% Asian, 84% Black). This degree of control has obvious value to experimenters seeking to maximize internal validity by manipulating stimulus features with precision.

Despite these benefits, however, several drawbacks may concern researchers. The first set of issues stems from consequences inherent to the morphing paradigm and the potential confounds researchers build into stimuli when they utilize morphs. These include the fact that morphing produces faces that are likely more physically attractive and less realistic than parent faces. Morphing works by melding pixels from the parent faces, thereby smoothing out imperfections and attenuating idiosyncratic facial features. The resulting faces are thus more “average” – by which we mean literally mathematically average, which has been robustly shown to be a strong predictor of attractiveness (Langlois & Roggmand, 1990). To mitigate this concern, researchers could conceivably compare morphed biracial stimuli to morphs made of two same-race faces, which would eliminate the confound of morphing. Morphed faces are also judged to look more “photoshopped” looking than the real parent faces used to generate them (Ma, Kantner, Benitez, et al., 2020). This is a problem when researchers compare images of real monoracial faces to artificial morphs, because the racial makeup of the faces and the degree of realism are perfectly confounded. Again, this concern could be allayed if researchers compare perceptions of same-race morphs to multi-race morphs (e.g., Chen et al., 2018; Kang, Plaks, & Remedios, 2015, Study 3; Pauker et al., 2018), but not all research has done so (e.g., Chen & Hamilton, 2012; Dickter & Kittel, 2012; Peery & Bodenhausen, 2008).

A second potential problem associated with using facial morphs to operationalize multiracialism is the so-called “emergent race phenomenon”. When researchers utilize facial morphs created from two monoracial parent faces, they assume that perceptually, the resulting multiracial stimulus will look like some combination of the parent faces; however, previous research demonstrates that facial morphs may be categorized as a different race or ethnicity altogether. Maclin, Maclin, Peterson, Chowdhry, and Joshi (2009) presented participants with Black–White morphed faces and observed that faces toward the center of the morph continuum were more likely to be categorized as Hispanic than either Black or White, even though neither parent face was Hispanic (see also Chen et al., 2018). Work by Nicolas, Skinner, and Dickter (2019) similarly shows that Black–White morphed faces frequently garner the labels Hispanic and Middle Eastern when participants are given an opportunity to categorize targets using open-ended responses. Although the emergent race phenomenon was first documented using morphed faces (Maclin et al., 2009), others have since demonstrated the effect using real faces (e.g., Chen et al., 2018; Nicolas et al., 2019), raising open questions about whether the emergent race effect poses a threat to validity or whether it is a consequence of appearing multiracial.

Finally, we contend that researchers who use facial morphs to operationalize multiracialism are limited in their ability to investigate questions related to classification concordance or discordance. Researchers within multiracial face perception generally veer away from the term accuracy (e.g., Chen & Hamilton, 2012), due in part to the complexities associated with conceptualizations of multiracialism discussed above. A significant portion of the existing multiracial face perception literature assesses concordance/discordance in categorizing multiracial targets by showing multiracial morphed faces to participants and asking them to categorize them by race (e.g., Chen & Hamilton, 2012; Gaither et al., 2019; Krosch, Berntsen, Amodio, Jost, & Van Bavel, 2013). However, we worry that inferences drawn from studies using morphs may be tautological: researchers engineer morphs to look biracial based on a priori notions of what multiracial individuals look like (i.e., an averaging of monoracial features), and then concordance is scored when participants’ judgments comport with researchers’ expectations, when in fact morphed faces have no real racial background. As an alternative to morphing, researchers might consider altering individual facial features to change perceptions in targets as multiracial (e.g., Ma, Kantner, Dunn, et al., 2020). For example, a researcher might adjust the nose shape or skin tone or even add freckles to a target in an attempt to create faces that match perceivers’ expectations of what multiracial faces look like.

Computer-generated multiracial faces are another example of artificial faces used in multiracial face perception research. Researchers have used computer-generated faces to study racial categorization and stereotyping of both monoracial (Gaither et al., 2019; Hugenberg & Bodenhausen, 2003) and biracial targets (e.g., Gaither et al., 2019; Pauker et al., 2009). These stimuli could conceivably be produced in any number of ways, but FaceGen Modeller (Singular Inversions, 2003) appears to be the most widely adopted software application used in the literature. FaceGen allows researchers to adjust various features, such as skin tone, facial feature, emotion expression, and emotional intensity of a base face to create unique faces. In general, computer-generated faces share many advantages (e.g., tight control) and disadvantages (e.g., artificiality) with morphed faces, with the possible exception of enhanced attractiveness, which has not been empirically tested as far as we are aware. It is also unclear whether computer-generated faces are subject to the emergent race phenomenon.

### Real faces

Somewhat ironically, researchers have rarely used real faces when studying multiracial face perception and we speculate that the general lack of available real multiracial face stimuli may explain why. Additionally, the research that has been conducted using real multiracial faces tends to utilize only a small number of target faces. Pauker and Ambady (2009; see also Pauker, 2009), for example, used a sample of 44 pictures of real Asian–White biracials to study race classification. These stimuli came from a high school yearbook and Pauker personally confirmed the racial self-identifications of these biracial individuals. Subsequently, Chen and Hamilton (2012) used eight real Black–White biracial individuals who were borrowed from Pauker et al. (2009). More recently, Iankilevitch, Cary, Remedios, and Chasteen (2020) curated a set of 13 Asian–White photos from student volunteers and Nicolas et al. (2019) included ten real Black–White multiracials in their research. Gaither et al. (2019) photographed 30 Black–White individuals for a series of studies examining how real versus computer-generated faces and response sets influence race essentialism. Of these 30 faces, 20 were deemed sufficiently racially ambiguous. Important for the current research, Gaither et al. (2019) find that real and computer-generated biracial faces differed on several key metrics. Compared to computer-generated faces, real faces were more likely to be categorized in line with hypodescent, required less time to be categorized, and when participants viewed real biracial faces they endorsed race essentialist beliefs to a greater extent than when they were shown computer-generated faces. Ultimately, a survey of the literature suggests that the multiracial face perception literature relies on very few real multiracial faces (for a review see Chen et al., 2020). Drawing conclusions from fairly small sets of stimuli can pose threats to both internal and external validity (Judd, Westfall, & Kenny, 2012). A second issue with real face stimuli concerns the availability of stimuli for broader use. Regarding this point, although Iankilevitch et al. (2020) reported that participants consented to the use of their photos for future research, we do not know the extent to which these images are available to researchers outside the lab. In the case of Pauker and Ambady (2009), the targets may not have given consent for their images to be used for experimental purposes and the researchers may not have permission to distribute their stimulus sets. Limited access to research stimuli of any sort – not just images of faces – poses challenges for direct replications, but also prohibits careful review of important methodological details (e.g., evaluating possible idiosyncrasies of the stimuli, assessing the stimuli for confounds, etc.), highlighting the value for publicly available research tools. A third important consideration is that the real multiracial face stimuli that have been developed by different researchers likely vary considerably. These stimulus sets may differ in terms of image resolution, lighting conditions, pose, face positioning, clothing, cropping, and a host of other highly relevant dimensions. The lack of standardization impedes researchers’ ability to sample across stimulus sets without introducing confounds.

The concerns we raise make clear the need for a database of freely accessible stimulus pool of real images of self-identified multiracial individuals. Indeed, we are not alone in this view, as Chen et al. (2020) have recently published a database of multiracial faces dubbed the American Multiracial Faces Database (AMFD). The AMFD includes 110 multiracial individuals who were photographed with both a neutral expression and a smile. Subsequently, subjective ratings of the targets were gathered (e.g., perceived dominance, trustworthiness, racial/ethnic prototypicality, masculinity/femininity, etc.). The current research seeks to further contribute to this effort by offering an additional resource for multiracial standardized face stimuli and related norming data. Below, we describe the development of the database and further discuss its unique contribution (General Discussion).

## The current research

The current paper introduces a free database containing images of real biracial individuals along with targets’ self-reported race and ethnic ancestry, norming data, and objective measurements in the form of an expansion of an existing and widely used resource – the Chicago Face Database (CFD; Ma et al., 2015). The CFD was published in 2015 and initially included images of 158 neutrally expressed Black and White male and female individuals. Since its initial publication, the CFD has been expanded to include 597 images of Asian, Black, Latino, and White males and females. In addition to digital images of real people, the CFD includes norming data and objective physical measures of the faces – all of which is available for free download at www.chicagofaces.org. Recruitment for the CFD was limited to self-identified monoracial individuals, and although norming data reveals variability in targets’ perceived racial and ethnic backgrounds, the racial backgrounds of the CFD targets is unknown. We chose to build upon the CFD for two reasons: to facilitate research protocols that might require images of real multiracial and monoracial face stimuli without confounding other aspects of the stimuli (e.g., background, resolution, clothing, lighting, etc.) and because at the time we conducted this research, the CFD was one of the most widely used face databases.

A secondary goal of the current research is to better understand the features that correspond with perceptions of multiracialism. Because previous research involving real multiracial faces has been so scant, very little is known about the physical properties and subjective perceptions of self-identified multiracial faces. To this end, we plan to examine the physical measurement and norming data to uncover the physical features that describe the natural variation among multiracial faces through factor analysis and explore associations between perceived multiracialism and subjective ratings of the faces using correlational analyses.

## Method

### Stimulus development

#### Target recruitment

Individuals were recruited from the greater Los Angeles area. We recruited students from California State University, Northridge using advertisements to the human subjects pool, printed fliers, and in-person solicitation. We also advertised to individuals on an online job forum. Participants were required to be between 18 and 60 years of age. Recruitment materials explicitly stated that we were, “looking for multiracial participants for research purposes”. Each individual who expressed interest in participating was then asked to provide the racial background information for their parents before they were permitted to participate in the study. Any participant whose racial background did not include mixed racial heritage were screened out of the study. As described earlier, we fully acknowledge the drawbacks associated with operationalizing multiracial in this way. However, using ancestry and self-identification aligns with the way that the United States government defines multiracialism in its Census and, as such, is likely consistent with how potential targets and perceivers conceive of multiracialism. Further, using ancestry and self-identification as selection criteria also does not preclude researchers’ ability to further select faces based on social consensus or racial ambiguity, whereas the reverse (i.e., recruiting based on social consensus or racial ambiguity) could yield a set of faces that are not necessarily multiracial based on ancestry or self-identification. The final sample included 88 multiracial individuals (62 female, 26 male). Within the sample, the majority of targets reported Asian and European ancestry of some kind. Target-level self-reports are available for evaluation as part of the datafile.

#### Photographing

The process for taking photographs was identical to the procedures used for collecting the CFD targets (Ma et al., 2015). First, participants provided written consent releasing their images to us for research purposes. The terms of this release are available at (www.chicagofaces.org). Next, participants changed into a heather grey t-shirt (or wore it over their normal clothing). Participants were then seated at a fixed distance from the camera and the height of the camera was adjusted to be at the participants’ eye level. We used a Nikon D90 mounted with a 50-mm1/8 f lens to capture the images. The photography studio was set up with a plain white backdrop and lighting conditions matching those used to produce the CFD images. Participants were photographed making neutral facial expressions, as well as various emotion expressions; however, we only include the neutral expressed images here. We plan to make the emotion expression faces available once we are able to process and norm them. The participants were photographed to include the shoulders. Participants were asked to square their shoulders and face toward the camera. Their heads were also adjusted to be straight, not tilted. Photographs were taken in high-resolution, raw format. The sessions lasted about 15–20 minutes and participants were compensated between $20 (students) and $100 (job forum recruits) for their time.

#### Stimulus standardization

In order to maintain consistency with the existing CFD images, we employed identical processing of the images. We selected one neutral image per target, sized at 4288 pixels (wide) × 2848 pixels (high). Images were downsized to 2444 pixels (wide) × 1718 pixels (high) and then edited using Adobe Photoshop. Target size was equated across images by fitting a 796 pixels (wide) × 435 pixels (high) rectangular guide mask over the targets’ core facial features, such that the mask met one or both of the following conditions: (1) the height of the mask matched the vertical distance between the lowest part of the inner brow and the top of the upper lip, or (2) the width of the rectangle matched the horizontal distance between the farthest visible extent of the cheek bones. Targets were placed against a pure white background and the coloring of the images was adjusted for warmth.

### Subjective ratings

We used the same format for obtaining norming data that was used when creating the CFD. Neutral expression images were presented to 499 participants who were recruited via Amazon’s Mechanical Turk. Raters included 240 females, 257 males, and two other/non-binary. The sample included 358 Whites, 46 Asians, 42 Blacks, 24 Latinos, 18 Biracial/Multiracial, 9 Native American, 1 Pacific Islander, and 1 participant who reported Other as their race. The average age of the sample was 36.31 (SD = 11.19) years old. For each rater, ten randomly selected multiracial targets were selected, presenting one at a time. For each target, raters first saw the target pictured and below were prompted to provide an estimation of the target’s age (“Estimate the approximate age of this person in years.”). Second, they saw the target on a new screen and were asked to categorize the target by race (“What race is this person?”). Raters could select Asian, Black, Hispanic/Latino, White, Biracial or Multiracial, or Other. If the rater selected either Biracial or Multiracial or Other, they were permitted to submit a text string providing more information about their judgment. Third, they saw the target on a new screen and were asked to provide a gender categorization (“What is this person’s gender?”). Raters could select male or female. Finally, raters saw the target on a new screen and were asked, “Now, consider the person pictured above and rate him/her on the following attributes.” Raters judged the targets in terms of how Threatening, Masculine, Feminine, Baby Faced, Attractive, Trustworthy, Happy, Angry, Sad, Disgusted, Surprised, Fearful/Afraid, and Unusual (would stand out in a crowd) they appeared using a 7-point Likert-type scale (1 = Not at all, 7 = Extremely). Rating the faces required approximately 20 minutes, and participants were compensated $1.26 in exchange.

### Objective measures

Finally, we took exhaustive physical measures of each target using Adobe Photoshop. Trained research assistants measured the median hair luminance, median eye luminance, median luminance of the face, nose width, nose length, lip thickness, face length, height and width of each eye, face width at the most prominent part of the cheek, face width at mouth, forehead length, distance between each pupil and the top of the head, distance between each pupil and the upper lip, chin length, length of cheek to chin for both sides of the face, and distance between pupils (see Ma et al., 2015 Table 1 for a list of all the measured features along with detailed descriptions of how each measure was made). Two different research assistants measured each face. The reliability of the measures was assessed by taking an average of the two measurements and an absolute difference score. Measures that had an absolute difference score equal to or greater than 20% of the average were flagged and a third research assistant measured the feature and resolved any discrepant measures. This procedure was sufficient to resolve all discrepant measures. We also used raw measures to compute composite measures of the face that commonly appear in face perception research (e.g., facial width-to-height ratio, face shape, nose shape, etc.; Blair & Judd, 2011; Zebrowitz & Collins, 1997).

**Table 1:** Correlation matrix showing associations between factor scores derived from physical measures and subjective ratings

	Mean	SD	α	Factor 1	Factor 2	Factor 3	Factor 4	Factor 5	Factor 6
Asian (proportion)	0.207	0.235		0.107	–0.015	–0.053	–0.154	.328^**^	0.043
Biracial or Multiracial (proportion)	0.103	0.081		0.076	–0.004	–0.144	0.164	–0.045	–0.031
Black (proportion)	0.209	0.313		–0.16	–0.041	0.17	.267^*^	–.618^**^	–.324^**^
Latino (proportion)	0.256	0.193		0.024	0.026	–0.102	0.027	0.11	.212^*^
White (proportion)	0.191	0.239		0	0.079	–0.001	–.306^**^	.406^**^	.251^*^
Other (proportion)	0.033	0.059		.241^*^	–0.122	–0.156	0.122	0.03	–0.119
Male (proportion)	0.309	0.451		.446^**^	–0.024	–0.034	–.296^**^	0.08	–.247^*^
Female (proportion)	0.691	0.451		–.446^**^	0.024	0.034	.296^**^	–0.08	.247^*^
Age (average)	27.357	4.082	0.996	0.001	–0.171	–0.158	–0.04	–0.043	–0.094
Angry	2.311	0.500	0.981	–0.078	0.035	0.154	–0.027	–.266^*^	–0.09
Attractive	3.635	0.717	0.992	–.268^*^	0.064	–0.186	0.148	0.108	0.156
Baby faced	2.617	0.606	0.985	0.121	0.068	0.173	0.028	.376^**^	0.167
Fear	2.015	0.278	0.92	–0.156	0.07	–0.104	0.187	–0.05	0.167
Feminine	3.944	1.441	0.998	–.450^**^	0.044	–0.048	.275^**^	–0.01	.275^**^
Happy	2.512	0.589	0.983	0.027	–0.005	–0.007	–0.015	.263^*^	0.021
Masculine	2.903	1.310	0.997	.458^**^	–0.07	0.022	–.264^*^	–0.029	–.285^**^
Sad	2.487	0.455	0.997	–0.063	–0.007	0.06	–0.072	–0.203	0.067
Surprised	1.880	0.233	0.997	0.007	0.045	–0.106	0.168	.216*	0.097
Threatening	2.104	0.401	0.997	0.076	–0.112	0.112	–0.035	–.284^**^	–0.151
Trustworthy	3.666	0.389	0.997	–0.018	–0.063	–0.103	0.142	.298^**^	0.195
Unusual	2.467	0.323	0.997	0.075	0.057	0.18	0.088	–0.206	–0.131

## Results

### Subjective ratings

First, we sought to establish the reliability of the norming data. Recall that because it would have been too onerous to ask raters to judge all 88 targets while maintaining the fidelity of the ratings, raters only judged a subset of ten faces each. This resulted in a data file that had large amounts of randomly missing data. As such, we used an estimation of interdependence procedure to compute reliability (Kenny & Judd, 1996; Judd & McClelland, 1998). This technique yielded estimates of the reliability of single items, which were then submitted to the Spearman–Brown Prophecy Formula. Reliabilities for each judgment is presented in Table 1. Because we have a large sample (*n* = 499), the interrater reliability is inflated. That said, subjective ratings of the type we measured tend to have high correspondence across raters per previous research (Kenny & Judd, 1996).

Next, we were interested in exploring how the subjective ratings of the targets were associated. To accomplish this, we first computed the means and standard deviations of the subjective ratings and used means to compute zero-order correlations. The resulting correlation matrix is presented in Table 2. There are far too many correlations among the various subjective ratings to discuss in the body of the paper, but we highlight a few associations that relate to biracial classifications. Although raters could categorize targets into multiple racial categories, it makes sense that there should be negative correlations among the proportions of racial categorizations a target receives (e.g., we expect an inverse relationship between the proportion of classifications of targets as White versus Black). We did see some evidence of these relationships among the monoracial categories; however, the proportion of classifications as biracial/multiracial only inversely related to classifications as White, *r*(86) = –.23, *p* = .03. More biracial targets appeared less White. Interestingly, this inverse relationship was not statistically significant among biracial/multiracial classifications and any of the other race classifications, as if to suggest that being biracial/multiracial means looking less White, but not necessarily less Asian, Black, Latino, etc. Second, we observed that targets who elicited more categorizations as biracial were also seen as more unusual, *r*(86) = .23, *p* = .03. Interestingly, the association between unusual and classifications as Black was also significant, *r*(86) = .27, *p* = .01; however, targets who were classified as Latino were generally seen as less unusual, *r*(86) = –.22, *p* = .04.

**Table 2:** Correlation matrix showing associations between subjective ratings

	1	2	3	4	5	6	7	8	9	10	11	12	13	14	15	16	17	18	19	20	21
1. Asian (proportion)	--																				
2. Biracial or Multiracial (proportion)	−0.2	--																			
3. Black (proportion)	−.453^**^	0.026	--																		
4. Latino (proportion)	−.227^*^	0.084	−.423^**^	--																	
5. White (proportion)	−0.161	−.227^*^	−.487^**^	−0.097	--																
6. Other (proportion)	0.081	−0.06	−0.184	0.154	−0.19	--															
7. Male (proportion)	.280^**^	0.056	−.256^*^	−0.063	0.055	0.15	--														
8. Female (proportion)	−.280^**^	−0.056	.256^*^	0.063	−0.055	−0.15	-1.000^**^	--													
9. Age (average)	−0.082	0.016	−0.041	0.103	0.008	0.153	−0.066	0.066	--												
10. Angry	−.358^**^	−0.038	.322^**^	−0.057	−0.016	0.017	−0.13	0.13	0.139	--											
11. Attractive	0.036	0.062	−0.192	.212^*^	0.04	−0.066	−.348^**^	.348^**^	−0.201	−.300^**^	--										
12. Baby faced	.266^*^	0.109	−.239^*^	−0.072	0.124	−0.205	.288^**^	−.288^**^	−.621^**^	−.384^**^	0.116	--									
13. Fear	−.213^*^	0.025	0.069	0.153	0.014	−0.103	−.243^*^	.243^*^	−0.051	.234^*^	−0.045	0.146	--								
14. Feminine	−.211^*^	−0.026	0.129	0.098	0.009	−0.163	−.945^**^	.945^**^	0	−0.019	.574^**^	−0.184	0.191	--							
15. Happy	0.171	−0.055	−.212^*^	0.101	0.077	−0.128	−0.02	0.02	−0.017	−.647^**^	.445^**^	.327^**^	−.321^**^	0.153	--						
16. Masculine	0.142	0.067	−0.132	−0.027	−0.006	0.156	.945^**^	−.945^**^	0.053	0.039	−.484^**^	0.155	−0.159	−.972^**^	−0.126	--					
17. Sad	−0.201	−0.029	0.138	0.059	−0.026	0.022	−0.137	0.137	0.064	.402^**^	−.302^**^	−0.098	.640^**^	0.033	−.577^**^	−0.026	--				
18. Surprised	−0.003	0.093	−0.178	0.147	0.095	−0.034	−0.029	0.029	0.036	−.218*	.186	.381**	.455**	.064	.417**	.005	.031	--			
19. Threatening	−.370^**^	−0.059	.337^**^	−0.002	−0.085	0.12	0.001	−0.001	.227^*^	.875^**^	−.434^**^	−.403^**^	.228^*^	−0.194	−.560^**^	.219*	.360^**^	−0.09	--		
20. Trustworthy	.272^*^	0.033	−.315^**^	0.116	0.068	−0.113	−0.048	0.048	−0.106	−.657^**^	.652^**^	.395^**^	−0.119	.248^*^	.752^**^	−0.193	−.368^**^	.393**	−.674^**^	--	
21. Unusual	−0.037	.232^*^	.265^*^	−.217^*^	−.217^*^	0.015	−0.11	0.11	−0.087	0.177	−0.108	0.117	0.175	0.06	−0.011	−0.028	0.082	.240*	0.187	−0.073	--

### Objective ratings

Next, we explored the objective measures of faces by conducting a factor analysis of the multiracial faces. We submitted median face luminance, face length, face width at cheeks, face width at mouth, face shape, heartshapedness, nose shape, lip fullness, eye shape, eye size, upper head length, midface length, chin length, forehead height, cheekbone height, cheekbone prominence face roundness, and facial width to height ratio to an exploratory factor analysis using a principal component analysis with varimax rotation. A six-factor solution explained 88.27% of the variance among the multiracial faces (see Table 3 for component matrix). To help uncover the latent construct underlying each factor, we correlated factor scores with subjective ratings (see Table 1). Factor 1 corresponded most strongly with face roundness and correlational analysis revealed moderate correlations with gender-related variables. Factor 1 explained 28.15% of variability among the faces and had an eigenvalue of 5.07. Factor 1 correlated positively with classifications as male, *r*(86) = .45, *p* < .001 and masculinity, *r*(86) = .46, *p* < .001, and negatively with classifications as female, *r*(86) = –.45, *p* < .001 and femininity, *r*(86) = –.45, *p* < .001. Factor 1 also negatively correlated with ratings of attractiveness, *r*(86) = –.27, *p* = .01. Factor 2 explained 20.61% of the variability and had an eigenvalue of 3.71. The construct underlying Factor 2 was less apparent. The measures that corresponded with Factor 2 most strongly were heartshapedness, upper head length, midface length, forehead height, and cheekbone prominence. No significant correlations between Factor 2 and any subjective measures emerged. Likewise, Factor 3 corresponded most strongly with fWHR and eye shape, but no correlations between Factor 3 and any of the subjective ratings were observed. Factor 4 explained 11.76% of the variability among the faces and had an eigenvalue of 2.12. Factor 4 appeared to represent eyes, as eye shape and eye size emerged as the strongest loadings for Factor 4. When we examined the correlations, we found weak but significant correlations between Factor 4 and gender and race. Factor 4 correlated positively with categorizations as Black, *r*(86) = .27, *p* = .01; categorizations as female, *r*(86) = .30, *p* = .005, and ratings of facial femininity, *r*(86) = .28, *p* = .01. Conversely, Factor 4 correlated negatively with categorizations as White, *r*(86) = –.31, *p* = .004; categorizations as male, *r*(86) = –.30, *p* = .005, and ratings of facial masculinity, *r*(86) = –.26, *p* = .01. Factor 5 explained 11.30% of variance among the faces and had a corresponding eigenvalue of 2.03. Factor 5 related to lip fullness and skin luminance and corresponded with categorizations as Black, *r*(86) = –.62, *p* < .001, categorizations as White, *r*(86) = .41, *p* < .001, and categorizations as Asian, *r*(86) = .33, *p* = .002. Interestingly, Factor 5 correlated with a host of subjective ratings, including facial anger, *r*(86) = –.27, *p* = .01, baby faced, *r*(86) = .38, *p* < .001, happiness, *r*(86) = .26, *p* = .01, threatening, *r*(86) = –.28, *p* = .007, and facial trustworthiness, *r*(86) = .30, *p* = .005. Finally, luminance, face width at cheeks, and chin length loaded on Factor 6. Factor 6 explained 10.12% of variability among faces and had an eigenvalue of 1.82. Categorizations as Black negatively related to Factor 6, *r*(86) = –.32, *p* = .002, but positively with categorizations as Latino, *r*(86) = .21, *p* = .05, and White, *r*(86) = .25, *p* = .02. Factor 6 also related weakly, but significantly with categorizations as female, *r*(86) = .25, *p* = .02 and facial femininity, *r*(86) = .28, *p* = .01. Factor 6 was weakly and negatively associated with categorizations as male, *r*(86) = –.25, *p* = .02 and facial masculinity, *r*(86) = –.29, *p* = .007.

**Table 3:** Factor analysis component matrix of physical face measures

	Factor 1	Factor 2	Factor 3	Factor 4	Factor 5	Factor 6
Median Luminance	0.138	–0.073	–0.061	–0.353	0.579	0.418
Face Length	–0.270	–0.725	–0.082	–0.206	–0.218	–0.163
Face Width Cheeks	0.641	–0.089	0.138	0.130	0.436	–0.476
Face Width Mouth	0.766	–0.531	–0.276	0.084	0.121	–0.169
Faceshape	0.667	0.417	0.174	0.236	0.466	–0.255
Heartshapeness	–0.590	0.643	0.414	–0.026	0.127	–0.105
Noseshape	0.666	–0.212	0.356	0.288	–0.342	0.084
Lipfullness	0.128	0.311	0.053	0.510	–0.645	–0.197
Eyeshape	–0.330	0.113	–0.549	0.620	0.158	0.272
Eyesize	–0.154	0.367	–0.484	0.664	0.201	0.251
Upperhead Length	–0.560	–0.672	0.255	0.210	0.264	–0.022
Midface Length	–0.060	0.602	–0.547	–0.382	0.183	–0.264
Chin Size	0.640	0.055	0.247	–0.354	0.072	0.453
Forehead Height	–0.560	–0.672	0.255	0.210	0.264	–0.022
Cheekbone Height	0.644	0.407	–0.078	–0.252	–0.346	0.162
Cheekbone Prominence	–0.401	0.719	0.467	0.028	0.231	–0.146
Face Roundness	0.883	–0.226	–0.228	0.176	0.206	–0.097
fWHR	0.444	0.106	0.614	0.403	0.092	0.298

## General discussion

The current research addresses a pressing need for stimuli in the multiracial face perception domain. Given recent trends in the literature and a growing multiracial population in the US and worldwide, it seems inevitable that more research related to multiracialism will be conducted. Here, we introduce a set of 88 multiracial stimuli along with self-reported ancestry, physical measurements, and norming data – all of which can be downloaded at www.chicagofaces.org. We emphasize that this work complements, rather than duplicates, the AMFD (Chen et al., 2020). Although both the current set of faces and the AMFD include a large diversity of real multiracial faces and subjective norming data, there are several unique contributions that set the current work apart. First, the current set of multiracial face stimuli are fully compatible with the existing CFD faces. Because the images were taken under the exact conditions as the previous set of CFD images and processed in an identical manner, researchers who require non-multiracial (i.e., Asian, Black, Latino, or White) faces for their research protocols can find faces in the existing database without worrying about potential confounds. Relatedly, a second unique contribution of the multiracial expansion to the CFD is the stringent standardization of the CFD image set. Targets were photographed seated in a standardized position relative to a tripod-mounted camera, with professional studio lighting that ensured consistent lighting conditions. The setup ensured that faces were consistently captured on a level plane, rather than from above or below. The addition of lighting and tripod resulted in well focused photographs that capture individual features with high fidelity. Additionally, targets were also photographed without extraneous accessories to ensure attention to the intended stimulus, rather than something extraneous to the face. A third unique contribution of the current face set is the available norming data. Whereas the AFMD includes subjective norming data for some dimensions we did not collect, the current set of faces were normed for unusualness, anger, fear, disgust, happiness, babyfacedness, threat, and unusualness. Additionally, we include comprehensive physical measurements of all faces. These measurement data are labor intensive and require specific software. They make it easy for researchers to select faces based on different physical dimensions or even use these measurements as variables in research protocols (see for example, Deska, Lloyd, & Hugenberg, 2018). Fourth, we recruited targets from a college campus as well as the broader community. This allowed us to capture a somewhat broader range of targets with respect to perceived age than those in the AFMD, who were primarily recruited from a college population. Finally, the targets included here were photographed making a variety of additional facial expressions and with direct and indirect gaze.

As we noted above, we suspect that a major reason researchers predominantly use artificial stimuli to represent multiracialism is the scarcity of real, research-grade multiracial face stimuli. We are encouraged to see others (Chen et al., 2020) recognize this need and work to fill the existing gap. With these new resources for multiracial face stimuli, researchers have increased flexibility and choice in their research, which we hope will afford greater experimental precision. And, we hope that the current face set encourages researchers to turn to real multiracial faces in addressing important questions regarding the perception, classification, and treatment of multiracial people.

In exploring the associations between multiracial classifications of a given target and some of the subjective measures, a few relationships stand out that we believe are worthy of unpacking further. We observed, for example, that faces that garnered higher rates of biracial/multiracial categorizations also tended to be rated as more unusual. We have observed this same relationship in other recent studies from our lab using different stimuli and samples (Ma, Kantner, Benitez, et al., 2020; Ma, Kantner, Dunn, et al., 2020). Speculatively, multiracial faces may be seen as unusual because they are literally less frequent in the population and thus are statistically unusual. Alternatively, these faces may be perceptually unusual in that they possess combinations of features that do not allow them to be categorized as monoracial. If the former hypothesis is true, we would expect that perceivers living in areas where there are more multiracials may not show this positive association, whereas those who are less likely to encounter multiracials should show even more of this effect. If the latter hypothesis is true, then we might predict that targets that have features that create more conflict between categories might be categorized as multiracial more often.

We also observed that targets who had a higher proportion of classifications as biracial/multiracial were at the same time less likely to be classified as White. This pattern is consistent with hypodescent, the tendency for individuals to be categorized as members of the socially subordinate racial or ethnic group (e.g., Ho et al., 2011) and this effect may be especially pronounced given the stimuli were real multiracial faces (Gaither et al., 2019). Moreover, given the predominantly White participant sample from which this association was derived (an issue we return to below), it is also possible that these judgments could be driven by ingroup overexclusion (Yzerbyt, Leyens, & Bellour, 1995). White perceivers may detect non-White ancestry from a face and be less likely to conceive of it as White. Alternatively, perceivers could simply recognize multiracial faces as “not White” (Chen, Moons, Hamilton, & Sherman, 2014).

The factor analysis results also yielded some interesting findings. For example, we observed that Factor 4 related to both perceptions of targets as Black as well as subjective ratings of femininity. This pattern stands in stark contrast to research describing race as gendered (e.g., Johnson, Freeman, & Pauker, 2012) in which Blackness tends to facilitate perceptions of faces as masculine. Future research may want to investigate the possibility that gender and race relate differently in multiracial faces, or whether this is merely the result of collapsing across many types of multiracial faces. Another set of correlations worth noting were the relationships between perceptions of targets as Black and subjective ratings of targets as appearing angry and threatening. This finding provides a conceptual replication of Hugenberg and Bodenhausen (2004), who show that implicitly measured prejudice predicted the tendency to categorize racially ambiguous targets as Black when they were making hostile facial expressions. Finally, as we suggested above, future research may seek to use objective facial metrics to engineer faces that possess certain features in order to produce multiracial faces that are high in social consensus. Analyses of objective data could prove useful in advancing this goal. Although the current analysis included all of the multiracial faces, breaking this analysis down into different racial subcategories might enable us to better identify some of the latent constructs associated with these measures.

Beyond supplying a free and convenient source for stimuli, these real multiracial faces enable researchers to examine a host of novel research questions regarding accuracy based on ancestry in multiracial face classification. This is significant, as previous research finds that racial, ethnic, and national misidentification can also correspond to greater experienced discrimination (Vargas, Winston, Garcia, & Sanchez, 2016) and lower socioeconomic status (Vargas, 2014). Incorrectly categorizing individuals by race can also gravely impact psychological well-being and physical health. Recent research by Albuja and colleagues found that denying biracial individuals one of their racial identities led to greater stress (Albuja, Gaither, Sanchez, Straka, & Cipollina, 2019a) and reductions in social belonging, which contributed to well-being (Albuja, Sanchez, & Gaither, 2019b; see also Franco & O'Brien, 2018). In addition to these direct consequences for multiracial individuals, work by Bratter and Gorman (2011) illustrates the importance of recognizing multiracial individuals for delivering healthcare. In their study, they analyzed data collected from the Centers for Disease Control and Prevention across a 7-year span with the aim of determining how best to incorporate multiracials into the health disparities research. Their analyses suggest that classifying multiracial individuals into monoracial categories or defining them based on their non-White heritage insufficiently captures documented health differences among groups. Instead, the data supported categorizing multiracial people into multiracial subcategories based on racial and ethnic heritage. Taken together, these findings place value on conceptually defining multiracialism based on ancestral background, as opposed to focusing on consensus of perceivers or relying on race specifying features.

These multiracial faces can also serve as a resource for cognitive psychologists interested in category learning and the mental representation of categories. Artificial stimuli are often used in categorization research because they can be constructed to vary along experimenter-defined dimensions, but a large set of stimuli from an ambiguous natural category such as multiracial faces provide the opportunity to study category learning within an ecologically valid domain in which the dimensionality of the items, and thus the strategies used by participants to classify them, may be less predictable (e.g., Soto & Ashby, 2019). Because multiracial faces are categorized at low rates of concordance, these stimuli afford investigations into the efficacy of category learning strategies (e.g., verbal/rule-based and non-verbal/information-integration; Ashby, Alfonso-Reese, & Waldron, 1998) and the impact of individual differences on the use of these strategies (e.g., DeCaro, Thomas, & Beilock, 2008; Little & McDaniel, 2015).

One of the limitations of the current research is the gender imbalance of stimuli. Our database includes three females for every male. Although efforts to recruit more males were made, we were unsuccessful in achieving more parity. Additionally, we did not carefully sample across different types of biracials. Regarding both of these weaknesses, we anticipate the possibility of adding more stimuli to the database. Since its initial publication, for example, we have grown the CFD significantly by adding more targets, facial expressions, and norming data. We view this as an organic resource that can continually grow to meet various research needs.

Although understanding how individual differences contribute to the perception of these multiracial targets was not a goal of the current research, we can imagine countless ways in which rater characteristics could affect the norming data we collected. Already, the field has established that biracial face perception corresponds with individual differences in Social Dominance Orientation (an individual difference characterizing people who show a preference for group-based hierarchy and inequity; Ho, Sidanius, Cuddy, & Banaji, 2013), political conservatism (Krosch et al., 2013), essentialist beliefs (an individual difference whereby people believe there are inherent differences among people due to race; Pauker & Ambady, 2009), internal motivation to control prejudice (Chen et al., 2014), perceived socioeconomic status of targets (Freeman, Penner, Saperstein, Scheutz, & Ambady, 2011), and perceived resource scarcity (Rodeheffer, Hill, & Lord, 2012). Cataloging various characteristics of the norming sample and understanding how these traits correlate with ratings could constitute avenues for future research, especially as we continue to grow the CFD.

## References

[CR1] Albuja AF, Gaither SE, Sanchez DT, Straka B, Cipollina R (2019). Psychophysiological stress responses to bicultural and biracial identity denial. Journal of Social Issues.

[CR2] Albuja AF, Sanchez DT, Gaither SE (2018). Fluid racial presentation: Perceptions of contextual “passing” among biracial people. Journal of Experimental Social Psychology.

[CR3] Albuja AF, Sanchez DT, Gaither SE (2019). Identity denied: Comparing American or White identity denial and psychological health outcomes among bicultural and biracial people. Personality and Social Psychology Bulletin.

[CR4] Ashby FG, Alfonso-Reese LA, Waldron EM (1998). A neuropsychological theory of multiple systems in category learning. Psychological Review.

[CR5] Binning KR, Unzueta MM, Huo YJ, Molina LE (2009). The interpretation of multiracial status and its relation to social engagement and psychological well-being. Journal of Social Issues.

[CR6] Blair IV, Judd CM (2011). Afrocentric facial features and stereotyping. The Science of Social Vision.

[CR7] Bratter JL, Gorman BK (2011). Does multiracial matter? A study of racial disparities in self-rated health. Demography.

[CR8] Chen JM (2019). An integrative review of impression formation processes for multiracial individuals. Social and Personality Psychology Compass.

[CR9] Chen JM, Hamilton DL (2012). Natural ambiguities: Racial categorization of multiracial individuals. Journal of Experimental Social Psychology.

[CR10] Chen JM, Moons WG, Gaither SE, Hamilton DL, Sherman JW (2014). Motivation to control prejudice predicts categorization of multiracials. Personality and Social Psychology Bulletin.

[CR11] Chen, J. M., Norman, J. B., & Nam, Y. (2020). Broadening the Stimulus Set: Introducing the American Multiracial Faces Database. *Behavior Research Methods,* 1-19. 10.3758/s13428-020-01447-8 32705658

[CR12] Chen JM, Pauker K, Gaither SE, Hamilton DL, Sherman JW (2018). Black+ White = Not White: A minority bias in categorizations of Black–White multiracials. Journal of Experimental Social Psychology.

[CR13] DeCaro MS, Thomas RD, Beilock SL (2008). Individual differences in category learning: Sometimes less working memory capacity is better than more. Cognition.

[CR14] Deska, J. C., Lloyd, E. P., & Hugenberg, K. (2018). Facing humanness: Facial width-to-height ratio predicts ascriptions of humanity. *Journal of Personality and Social Psychology, 114*(1), 75. 10.1037/pspi0000110 28846003

[CR15] Dickter CL, Kittel JA (2012). The effect of stereotypical primes on the neural processing of racially ambiguous faces. Social Neuroscience.

[CR16] Farley R (2001). Identifying with multiple races. Report 01-491.

[CR17] Franco MG, O'Brien KM (2018). Racial identity invalidation with multiracial individuals: An instrument development study. Cultural Diversity and Ethnic Minority Psychology.

[CR18] Freeman JB, Penner AM, Saperstein A, Scheutz M, Ambady N (2011). Looking the part: Social status cues shape race perception. PLoS ONE.

[CR19] Gaither SE (2015). “Mixed” results: Multiracial research and identity explorations. Current Directions in Psychological Science.

[CR20] Gaither SE, Chen JM, Pauker K, Sommers SR (2019). At face value: Psychological outcomes differ for real vs. computer-generated multiracial faces. The Journal of Social Psychology.

[CR21] Gaither SE, Sommers SR, Ambady N (2013). When the half affects the whole: Priming identity for biracial individuals in social interactions. Journal of Experimental Social Psychology.

[CR22] Harris DR, Sim JJ (2002). Who is multiracial? Assessing the complexity of lived race. American Sociological Review.

[CR23] Hitlin S, Scott Brown J, Elder GH (2006). Racial self-categorization in adolescence: Multiracial development and social pathways. Child Development.

[CR24] Ho, A. K., Kteily, N. S., & Chen, J. M. (2020). Introducing the Sociopolitical Motive× Intergroup Threat Model to Understand How Monoracial Perceivers’ Sociopolitical Motives Influence Their Categorization of Multiracial People. *Personality and Social Psychology Review,* 1088868320917051. 10.1177/1088868320917051 32449637

[CR25] Ho AK, Sidanius J, Cuddy AJ, Banaji MR (2013). Status boundary enforcement and the categorization of black–white biracials. Journal of Experimental Social Psychology.

[CR26] Ho AK, Sidanius J, Levin DT, Banaji MR (2011). Evidence for hypodescent and racial hierarchy in the categorization and perception of biracial individuals. Journal of Personality and Social Psychology.

[CR27] Hugenberg, K., & Bodenhausen, G. V. (2003). Facing prejudice: Implicit prejudice and the perception of facial threat. *Psychological Science, 14*(6), 640–643. 10.1046/j.0956-7976.2003.psci_1478.x 14629699

[CR28] Hugenberg K, Bodenhausen GV (2004). Ambiguity in social categorization: The role of prejudice and facial affect in race categorization. Psychological Science.

[CR29] Iankilevitch, M., Cary, L. A., Remedios, J. D., & Chasteen, A. L. (2020). How do multiracial and monoracial people categorize multiracial faces? *Social Psychological and Personality Science*. *11*(5), 688–696.

[CR30] Singular Inversions. (2003). *FaceGen Modeller 3*. Toronto, ON Canada: Ver, 3.

[CR31] Johnson KL, Freeman JB, Pauker K (2012). Race is gendered: How covarying phenotypes and stereotypes bias sex categorization. Journal of Personality and Social Psychology.

[CR32] Judd CM, McClelland GH (1998). Measurement. Handbook of Social Psychology.

[CR33] Judd CM, Westfall J, Kenny DA (2012). Treating stimuli as a random factor in social psychology: A new and comprehensive solution to a pervasive but largely ignored problem. Journal of Personality and Social Psychology.

[CR34] Kang SK, Plaks JE, Remedios JD (2015). Folk beliefs about genetic variation predict avoidance of biracial individuals. Frontiers in Psychology.

[CR35] Kenny DA, Judd CM (1996). A general procedure for the estimation of interdependence. Psychological Bulletin.

[CR36] Krosch AR, Berntsen L, Amodio DM, Jost JT, Van Bavel JJ (2013). On the ideology of hypodescent: Political conservatism predicts categorization of racially ambiguous faces as Black. Journal of Experimental Social Psychology.

[CR37] Langlois JH, Roggmand LA (1990). Attractive faces are only average. Psychological Science.

[CR38] Little JL, McDaniel MA (2015). Individual differences in category learning: Memorization versus rule abstraction. Memory & Cognition.

[CR39] Ma DS, Correll J, Wittenbrink B (2015). The Chicago face database: A free stimulus set of faces and norming data. Behavior Research Methods.

[CR40] Ma, D. S., Kantner, J., Dunn, S., & Benitez, J. (2020). *Are biracials a cognitively distinct category: Mapping biracials in psychological space*. Unpublished manuscript.

[CR41] Ma, D. S., Kantner, J., Benitez, J., & Dunn, S. (2020). *Face-off: Do morphed stimuli represent real multiracial faces*. Unpublished manuscript.

[CR42] MacLin OH, MacLin MK, Peterson D, Chowdhry O, Joshi P (2009). Social psychophysics: Using psychophysics to answer “social” questions with PsychoPro. Behavior Research Methods.

[CR43] Nicolas G, Skinner AL, Dickter CL (2019). Other than the sum: Hispanic and Middle Eastern categorizations of Black–White mixed-race faces. Social Psychological and Personality Science.

[CR44] Pauker, K. (2009). *Not so black and white: The impact of motivation on memory for racially ambiguous faces*. (Doctoral dissertation). Retrieved from ProQuest Dissertations & Theses Global database. (Accession Order No. AAT 3354706).

[CR45] Pauker K, Ambady N (2009). Multiracial faces: How categorization affects memory at the boundaries of race. Journal of Social Issues.

[CR46] Pauker K, Carpinella CM, Lick DJ, Sanchez DT, Johnson KL (2018). Malleability in biracial categorizations: The impact of geographic context and targets’ racial heritage. Social Cognition.

[CR47] Pauker K, Weisbuch M, Ambady N, Sommers SR, Adams RB, Ivcevic Z (2009). Not so black and white: Memory for ambiguous group members. Journal of Personality and Social Psychology.

[CR48] Peery D, Bodenhausen GV (2008). Black+ White= Black: Hypodescent in reflexive categorization of racially ambiguous faces. Psychological Science.

[CR49] Phinney J (1992). The Multigroup Ethnic Identity Measure: A new scale for use with adolescents and young adults from diverse groups. Journal of Adolescent Research.

[CR50] Phinney JS, Alipuria LL, Crisp RJ, Hewstone M (2006). Multiple social categorization and identity among multiracial, multiethnic, and multicultural individuals: Processes and implications. Multiple social categorization: Processes, models and applications.

[CR51] Remedios JD, Chasteen AL (2013). Finally, someone who “gets” me! Multiracial people value others’ accuracy about their race. Cultural Diversity and Ethnic Minority Psychology.

[CR52] Rodeheffer CD, Hill SE, Lord CG (2012). Does this recession make me look black? The effect of resource scarcity on the categorization of biracial faces. Psychological Science.

[CR53] Roth WD (2016). The multiple dimensions of race. Ethnic and Racial Studies.

[CR54] Serre, D., & Pääbo, S. (2004). Evidence for gradients of human genetic diversity within and among continents. *Genome research, 14*(9), 1679–1685. 10.1101/gr.2529604 PMC515312 15342553

[CR55] Skinner AL, Nicolas G (2015). Looking Black or looking back? Using phenotype and ancestry to make racial categorizations. Journal of Experimental Social Psychology.

[CR56] Soto FA, Ashby FG (2019). Novel representations that support rule-based categorization are acquired on-the-fly during category learning. Psychological Research.

[CR57] Spencer MS, Icard LD, Harachi TW, Catalano RF, Oxford M (2000). Ethnic identity among monoracial and multiracial early adolescents. The Journal of Early Adolescence.

[CR58] Strom MA, Zebrowitz LA, Zhang S, Bronstad PM, Lee HK (2012). Skin and bones: The contribution of skin tone and facial structure to racial prototypicality ratings. PLoS ONE.

[CR59] Townsend SS, Fryberg SA, Wilkins CL, Markus HR (2012). Being mixed: Who claims a biracial identity?. Cultural Diversity and Ethnic Minority Psychology.

[CR60] Udry JR, Li RM, Hendrickson-Smith J (2003). Health and behavior risks of adolescents with mixed-race identity. American Journal of Public Health.

[CR61] Vargas ED, Winston NC, Garcia JA, Sanchez GR (2016). Latina/o or Mexicana/o? The relationship between socially assigned race and experiences with discrimination. Sociology of Race and Ethnicity.

[CR62] Vargas N (2014). Off white: Colour-blind ideology at the margins of whiteness. Ethnic and Racial Studies.

[CR63] Woo M, Austin SB, Williams DR, Bennett GG (2011). Reconceptualizing themeasurement of multiracial status for health research in the United States. Du Bois Review: Social Science Research on Race.

[CR64] Yudell M, Roberts D, DeSalle R, Tishkoff S (2016). Taking race out of human genetics. Engaging a century-long debate about the role of race in science. Science.

[CR65] Yzerbyt VY, Leyens JP, Bellour F (1995). The ingroup overexclusion effect: Identity concerns in decisions about group membership. European Journal of Social Psychology.

[CR66] Zebrowitz LA, Collins MA (1997). Accurate social perception at zero acquaintance: The affordances of a Gibsonian approach. Personality and Social Psychology Review.

